# Primary Culture of Adult Rat Heart Myocytes

**DOI:** 10.3791/1308

**Published:** 2009-06-16

**Authors:** Xianghua Xu, Henry M. Colecraft

**Affiliations:** Department of Physiology and Cellular Biophysics, Columbia University; Department of Pharmacology, Columbia University

## Abstract

Cultured primary adult rodent heart cells are an important model system for cardiovascular research.  Nevertheless, establishment of robust, viable cultured adult myocytes can be a technically challenging, rate-limiting step for many researchers. Here we described a protocol to obtain a high yield of adult rat heart myocytes that remain viable in culture for several days.  The heart is isolated and perfused with collagenase and protease under low Ca^2+^ conditions to recover single myocytes.  Ca^2+^-tolerant cells are obtained by stepwise increases in extracellular Ca^2+^ concentration in three subsequent wash steps.  Cells are filtered, resuspended in culture medium, and plated on laminin coated slips. Cultured myocytes obtained using this protocol are viable for up to four days and are suitable for most experiments including electrophysiology, biochemistry, imaging and molecular biology.

**Figure:**
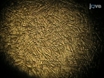


## Protocol

### Part I. Preparation for surgery

The day before the rat surgery, make certain that all solutions are made, but do not add any enzymes yet. Buffers A, B and the wash buffer should all be filtered for sterility prior to storage. The buffers should all be stored at 4 °C.
6-well tissue culture plates must be plated with 10-20 μg/mL laminin in 2 mL 1XSFM the day before as well, and should be stored at 37 °C in a CO_2_ incubator.
On the day of the surgery, prepare the surgical instruments by sterilizing the clamp, scissors, and forceps with 70 % ethanol and let them dry on paper towel.
A 1 mL syringe filled with 0.5 mL Heparin Solution (90 U/ml) is made ready.
Now it is good practice to wash the perfusion apparatus by running through 70% ethanol, using the peristaltic pump in reverse. Once finished rinsing with ethanol, rinse the apparatus with double distilled water, and finally rinse with Buffer A. The flow through is collected in a beaker placed in a 37  °C water bath.
To prepare the cleaned perfusion apparatus, fill the right syringe tube with 40 mL of Buffer A and the left syringe tube with 40 mL of Buffer B.
Prime the perfusion tubing to the tip of the catheter by allowing Buffer A to flow through the apparatus. Refill to the buffer A syringe to the 40 mL level. Make sure there are no air bubbles trapped in the tubing after this step.
Now, close the buffer A syringe stopcock valve and repeat the process with Buffer B such that the perfusion tubing is now primed with Buffer B.
Finally, the perfusion apparatus is left with the Buffer B stopcock valve open, but with the flow stopped using the regulator on the IV line.
Discard the buffer flow-though in the collecting beaker.
The last thing to do before the surgery is to add the required enzymes to your stock solution, and then filter the enzyme solution just as the other solutions were filtered the day before. Once filtered, this solution can be left at room temperature.

### Part II. The Rat heart dissection

Following standard protocol euthanize the rat with Isoflurane in a gas chamber.
Sterilize the incision area on the thorax with 70% ethanol.
Open chest using scissors and expose heart.
Inject left ventricle with 0.5 mL Heparin solution (90 U/mL).
Remove heart with large portion of aorta intact, place in ice cold Buffer B.

### Part III. Myocytes isolation

A typical rat heart should yield a high fraction of rod-shaped myocytes (as opposed to dead rounded cells) if the following procedure is properly adhered to*.*
To begin, clamp aorta to the catheter. The tip of the catheter should not be pushed too far into the heart to ensure good perfusion of the heart through the coronary artery.
Once clamped, tie the aorta to the catheter with a suture.
Next, open the IV line regulator to allow a fast drip rate (~60 drops/min) and allow Buffer B to wash out the heart.
During Buffer B wash, use the small scissors and forceps to remove the atria and any fatty or lung tissue clinging to the heart.
After Buffer B is finished, switch the flow to Buffer A.
While Buffer A is allowed to flow for 5 minutes, several small tasks should be quickly performed.
First, warm the Enzyme Solution in a 37 °C water bath.
Finally, when Buffer A is depleted from the syringe (but still present in the tubing), load   50 mL of the Enzyme Solution in syringe A of the perfusion apparatus
It is now necessary to discard all the flow-through from the collection beaker in the water bath (by pipetting), until the Enzyme Solution perfuses heart.
As soon as the Enzyme Solution perfuses the heart, activate the peristaltic pump which is set up to transfer the enzyme solution from the collecting beaker to replenish syringe A.
Allow the enzyme solution to flow through the heart for 10 min. As the heart digests, it begins to look bloated.
Add 37.5 μL of 0.1 M CaCl_2_ to the enzyme solution in syringe A to give an effective concentration of 0.1 mM Ca^2+^.
After 10 minutes increase the concentration of calcium to 0.2 mM by adding an additional 50 μL of 0.1 M CaCl_2_ to syringe A and let the perfusion proceed for a further 10 minutes.
Cut off the ventricles and transfer to a small sterile beaker containing 20 mL Enzyme Solution.
In the beaker increase the Calcium concentration to 0.4 mM by adding 40 more μL of 0.1 M CaCl_2_.
Gently mince the heart into 10 or more pieces with a pair of small sterile scissors.
Incubate the beaker for 5 minutes at 37 °C with gentle rocking.
Add 40 mL of 0.1 M CaCl_2_ to give effective concentration of 0.6 mM Ca^2+^ and gently triturate the heart pieces with a plastic transfer pipet 3-5 times.
After incubating for an additional 5 minutes at 37  °C, add 40 mL of 0.1 M CaCl_2_  and gently triturate as before.
Separate digested single myocytes from non-digested connective tissue using filtration through a sterile 500 μm mesh.
Allow the cells to settle in a 50 ml tube for 10 minutes at room temperature.
Discard supernatant with a transfer pipet.
Gently resuspend cells in Wash Buffer #1 and allow the cells to settle for 10-20 min at room temperature.
While the cells are settling, take a small aliquot, and use this time as an opportunity to assess the quality and viability of the cells in suspension under an inverted microscope. A good preparation will have a high proportion (>80%) of rod like cells with crisp striations.
Once the cells have settled, discard the supernatant and gently resuspend cells in Wash Buffer #2. Let the cells settle at room temperature, which should again take 10-20 minutes, and discard the supernatant.
Repeat the washing process once more with Wash Buffer #3, and the cells will be ready for culture.

### Part IV. Myocyte cell culture

Gently resuspend cells in 5% Serum Media. Before transferring the cells to tissue culture plates, wash the plates with 1x SFM.  Transfer the cells at a desired density and incubate in a CO_2_ tissue culture incubator for 3-5 hours.
Switch the media to 1x SFM.
Cells can be cultured for up to four days and used as required for experiments.

### Part V. Representative Results/Outcome

There should be more than 70% live heart myocytes under inverted microscope when the protocol is done correctly.


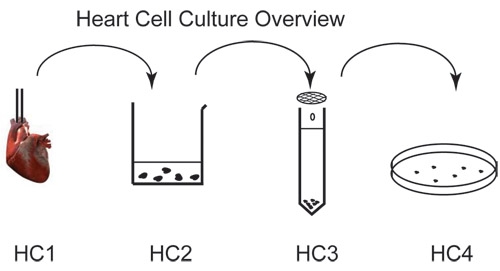


**Table 1: **Solution recipe for 1 L** 10X Ca^2+^-free KH Buffer**:

**Table:** 

	mass	Final Concentration
NaCl	68.96 g	1180 mM
KCl	3.58 g	48 mM
HEPES	59.58 g	250 mM
K_2_HPO_4_	2.85 g	12.5 mM
MgSO_4_	3.08 g	12.5 mM

**Table 2:** Solution recipe for 1 L **Buffer A:**Add 1.98 g glucose to 100 mL 10X KH buffer and bring to a final volume of 1 L. Adjust osmolarity to near physiology condition (~300) with glucose. Bring to pH 7.4 with NaOH.

**Table:** 

-	Mass	Final Concentration
10X KH Buffer	100 mL	100 ml
Glucose	1.98 g	11 mM
Bring to 1 L		
Bring to pH 7.4 with NaOH		
Adjust osmolarity to near physiological condition(~300) with glucose.

**Table 3:** Solution recipe for **EnzymeBuffers**

**Table:** 

	mass	Final Concentration
Collagenase Type II	25 mg	0.05% (w/v)
Protease XIV	10 mg	0.02% (w/v)
BDM	0.025 g	5 mM
Carnitine	0.020 g	2 mM
Taurine	0.031 g	5 mM
Glutamic Acid	0.020 g	2 mM
0.1 M CaCl_2_	12.5 uL	25 uM
Buffer A		Fill to 50 ml

**Table 4: **Solution recipe for 25X BDM/Taurine/BSA (B/T/B)

**Table:** 

	mass	Final Concentration
BDM	0.076 g	125 mM
Taurine	0.094 g	125 mM
BSA		0.1% (w/v)
Buffer A		Fill to 6 mL

**Table 5:** Solution recipe for **Buffer B **and** Wash Buffers**

**Table:** 

**Buffer:**	**B**	**#1**	**#2**	**#3**
**Add:**				
0.5 M CaCl_2_	0.4 mL	0.1 mL	0.125 mL	0.15 mL
**Final concentration of CaCl_2_**	1 mM	1 mM	1.25 mM	1.5 mM
25X B/T/B	---	2 mL	2 mL	2 mL
Buffer A to final volume	200 mL	50 mL	50 mL	50 mL

**Table 6:** Solution Recipe for Culturing Media

**Table:** 

**2X Serum-free Media (SFM)**		
****		**Final Concentration**
**(after dilution)**
Carnitine	0.099 g	10 mM (5 mM)
Taurine	0.063 g	10 mM (5 mM)
Creatine	0.075 g	10 mM (5 mM)
Antibiotic/Antimycotic	0.5 ml	1% (0.5%) (v/v)
Media 199	45 mL	Fill to 50 mL
		
**1X SFM**		
		**Final Concentration**
2X SFM	25 mL	1X
Media 199		Fill to 50 mL
		
**1X 5% Serum Media**		
		**Final Concentration**
2X SFM	25 mL	1X
FBS	2.5 mL	5% (v/v)
Media 199		Fill to 50 mL

## Discussion

A critical step is the speed with which the isolated heart is hung up on the perfusion system.  The length of the enzymatic digestion period may be a little different for each rat. The adjustment depends on how soft the heart becomes after the regular period of digestion. The slowly recovery of Ca^2+^ after enzyme digestion is essential for obtaining Ca^2+^-tolerant healthy cells.

For guinea pig, the protocol is the same except hyaluronidase is used instead of Protease XIV. Typically, we find the that initial percentage of living cells is lower for guinea pig compared to rat, although they survive just as long in culture.
